# Do proteolytic cascades exist in plants?

**DOI:** 10.1093/jxb/erz016

**Published:** 2019-02-18

**Authors:** Judith K Paulus, Renier A L Van der Hoorn

**Affiliations:** The Plant Chemetics Laboratory, Department of Plant Sciences, University of Oxford, Oxford, UK

**Keywords:** Peptidase, processing, prodomain cleavage, protease activation, proteinase, proteolytic cascade, signalling

**Proteolytic cascades are hierarchical sets of proteases that activate each other by proteolytic cleavage. Textbook examples are Ser proteases regulating blood coagulation and caspases regulating apoptosis. Many additional proteolytic cascades have been described in animal biology. In plants, however, knowledge on proteolytic cascades is fragmentary. Some plant proteases require non-self processing to become active, and vacuolar processing enzymes, subtilase-like, and papain-like proteases have been implicated in proteolytic cascades. We discuss these examples against four criteria that are set for proteolytic cascades in animal science and conclude that proteolytic cascades are likely to occur in plants but remain to be characterized.**

## What is a proteolytic cascade?

**Fig. 1.**
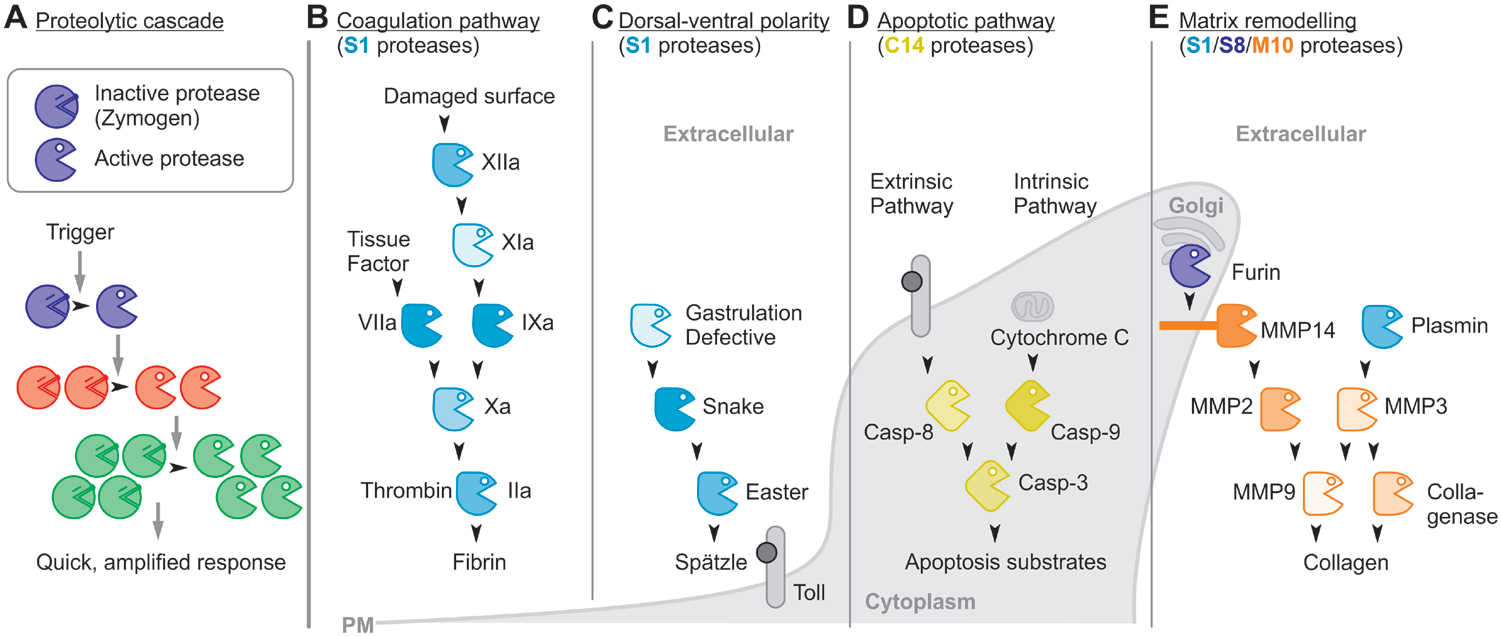
Textbook examples of proteolytic cascades. (A) General concept of a proteolytic cascade. (B–E) Simplified presentation of textbook examples of proteolytic cascades in animals. Only the active proteases are shown and feedback regulations are omitted. PM, plasma membrane. (B) S1 Ser proteases (Factors II–XII) regulating the blood coagulation pathway. (C) S1 Ser proteases regulating dorsal–ventral polarity during fruit fly embryogenesis. (D) C14 Cys proteases (caspases) regulating apoptosis. (E) S1, S8, and M10 proteases regulating matrix remodelling.

A proteolytic cascade consists of proteases that activate other proteases by processing (Fig. 1A). Most proteases are produced as zymogens carrying an N- or C-terminal inhibitory prodomain that folds into the active site cleft, often in an orientation opposite to that of a substrate (Verma *et al.*, 2016). These inhibitory prodomains prevent premature activity of the proteases. Prodomains also contribute to the folding of the proteases (Bryan, 2002) and often contain signals for subcellular targeting (McIntyre *et al.*, 1994; Huete-Pérez *et al.*, 1999).

Some proteases activate themselves by cleaving between the prodomain and the protease domain in a mono- or bimolecular manner (Rozman *et al.*, 1999; Turk *et al.*, 2000). This cleavage is frequently induced by a conformational change caused by a change in pH (Jerala *et al.*, 1998; Feliciangeli *et al.*, 2006; Meyer *et al.*, 2016; Zauner *et al.*, 2018). Other proteases, however, are activated by other proteases, creating additional regulatory control over protease activity. These proteolytic signalling cascades are fast, and quickly amplify the signal, taking advantage of constitutively expressed and highly selective proteases. Proteolytic cascades are common in the animal kingdom. We will discuss a few of the textbook examples and contrast this with the limited knowledge on proteolytic cascades in plants.

## Textbook examples of proteolytic cascades

Proteolytic cascades have been studied by medical scientists for a long time. One textbook example is the blood coagulation pathway, regulated by clotting factors, preventing blood loss upon injury through the rapid formation of a blood clot. Clotting factors are extracellular Ser proteases from the S1 family (MEROPS database, Rawlings *et al.*, 2018) that activate each other consecutively (Fig. 1B). The clot is formed when thrombin (FIIa, activated Factor II), the final protease of the cascade, cleaves fibrinogen to generate fibrin, which cross-links platelets to seal off injured vessels. The coagulation response is triggered by two parallel, merging proteolytic cascades involving S1 Ser proteases. One cascade is triggered by the release of tissue factor upon injury, which then forms a complex with the Ser protease FVII, activating it. FVIIa then activates protease FX. In parallel, upon contact with negatively charged surfaces of, for example, collagen, additional proteases FXII, FXI, and FIX are sequentially activated, leading to activation of FX by FIXa. FXa finally activates thrombin that cleaves fibrinogen to fibrin, allowing blood clot formation (Amara *et al.*, 2008). Tight regulation of these two merging proteolytic cascades occurs at multiple levels by additional cofactors, proteases, and inhibitors, and includes feedback regulation by thrombin itself. This proteolytic cascade causes a rapid, temporal, and localized response with a massive amplification of the initial signal.

Ser protease cascades also act in arthropods, for instance in haemolymph clotting and dorsal–ventral fate determination during fruit fly embryogenesis. Fruit fly embryogenesis involves three S1 Ser proteases (GD, Snake, and Easter) that act on each other to release Spätzle, the ligand for the Toll receptor (Fig. 1C; Cho *et al.*, 2012). All these cascades share several features. They have a functional core of essential Ser proteases that undergo sequential zymogen activation, resulting in processing of a terminal substrate. It is thought that these cascades are derived from a single ancestral protease cleaving the terminal substrate. Levels of regulation were subsequently added as upstream regulatory proteases (Krem and Di Cera, 2002). This hypothesis is supported by the observation that the terminal Ser protease is the most conserved protease between different proteolytic cascades (Krem and Di Cera, 2002).

However, proteolytic regulation through cascades is not exclusive to Ser proteases. Another important textbook example are caspases that act in inflammation and programmed cell death (PCD)-related processes such as apoptosis. Caspases are C14-family Cys proteases that cleave after Asp (hence ‘caspases’). Executor caspase-3 can be activated by initiator caspase-8 in the extrinsic pathway or by initiator caspase-9 in the intrinsic pathway, ultimately leading to apoptosis (Fig. 1C).

A single proteolytic cascade can also involve proteases from different protease families and act over different cellular compartments. For instance, signalling pathways to control remodelling of the extracellular matrix in mammals involve matrix metalloproteases (MMPs, M10 family) and proteases from the S1 and S8 families. MMPs are expressed at low levels in most tissues, and MMP expression and activation are tightly regulated (Brinckerhoff and Matrisian, 2002). Ser proteases furin and plasmin are activator proteases of the MMP cascade (Fig. 1D). Plasmin (trypsin-like S1 family) activates several MMPs, including MMP3 (stromelysin-1) which activates collagenase (M10 family) and MMP9 (gelatinase B) (Brinckerhoff and Matrisian, 2002). On the other hand, furin (subtilisin-like S8 protease residing in the Golgi) can activate MMP11 (stromelysin-3) and MMP14 (also called MT1-MMP) (Pei and Weiss, 1995; Santavicca *et al.*, 1996; Sato *et al.*, 1996). MMP14 is membrane anchored to focus MMP activity to the cell surface. MMP14 dimers interact with MMP2 (gelatinase A) and the tissue inhibitor of metalloproteases-2 (TIMP-2) in an orchestrated series of cell surface events that result in the activation of MMP2 (Itoh *et al.*, 2001), which then can also activate MMP9. Further intensive research in cancer biology revealed that MMP activation by other MMPs, furin, and cathepsin-B and -G is controlled through a complex, fine-tuned interactive protease network, rather than a simple proteolytic cascade (Mason and Joyce, 2011).

## Four criteria for proteolytic cascades

The examples discussed above illustrate that proteolytic cascades can involve different protease families, act at different subcellular locations, and regulate different biological processes. Nevertheless, despite their differences, all these textbook examples share common criteria. Here we outline four criteria shared by proteolytic cascades using the hypothetical cascade consisting of protease A processing protease B. Proteases A and B are part of a proteolytic cascade.

(1) ** Protease A activates protease B.** Other cleavage events, for example the degradation or inactivation of protease B by protease A, do not qualify as a proteolytic cascade.
(2) ** Proteases A and B should be co-localized**. Although co-localization of the proteases before or after this event is not mandatory, co-localization at the moment of processing is essential, for example if a protease is released from one compartment to activate a protease in another compartment.
(3) ** Protease B is the substrate of protease A *in vitro***. This means that the protease B precursor (pro-B) contains a cleavage site that matches the known specificity of protease A. This also implies that the modification of this sequence by targeted mutagenesis would lead to a protease B precursor that cannot be activated.
(4) ** Processing of protease B depends on protease A.** Protease B activation should be absent in mutant plants lacking protease A or the trigger to activate protease A, and these plants should accumulate the precursor of protease B.

## Proteolytic cascades in plants

Several publications speculate about plant proteases acting in cascades. We now critically review these cases against the four criteria summarized above. These cases are summarized in Table 1 and discussed below.

**Table 1.** Proteolytic cascades in plants.

Plant species	Tomato	*At*	Bean	*At*	Poplar	Barley
Protease A:	MMP	γVPE	VmPE-1	VPE	MC9	RD21
Protease B:	P69B	AtCPY	SH-EP	RD21	RD21	ALP
(1) A would activate B	N	N^*a*^	Y	Y	ND	Y
(2) A and B co-localize	Y	Y	Y	Y	Y^*a*^	Y
(3a) A cleaves B *in vitro*	Y	ND	Y	ND	ND	Y
(3b) ProB has cleavage site of A	Y	Y	Y	N^*a*^	N^*a*^	ND
(3c) Uncleavable ProB mutant	ND	ND	ND	ND	ND	ND
(4a) Without A, B is not processed	Y	Y	ND	N	Y	ND
(4b) Without A, ProB accumulates	Y	Y	ND	N	N^*a*^	ND

N, no; Y, yes; ND, not determined; *At*, *Arabidopsis thaliana.*

^*a*^ As reasoned in this Viewpoint.

### Do MMPs activate P69B in tomato?

MMPs are extracellular proteases from the M10 family that are often anchored in the cell membrane. In animals, MMPs process each other in a proteolytic cascade (see above and Fig. 1E). Plants also have MMPs, but most of these are uncharacterized (Marino and Funk, 2012). A study on two MMPs from tomato (Sl2- and Sl3-MMP) revealed that their activity is similar to that of their mammalian counterparts (Zimmermann *et al.*, 2016). Importantly, *Sl2/3-MMP* silencing caused cell death extending along the stem and into the leaves (Zimmermann *et al.*, 2016). These *Sl2/3-MMP*-silenced plants also accumulate P69B, an S8-family subtilase known to accumulate extracellularly upon biotic stress (Jordá *et al.*, 1999). The accumulation of P69B upon *Sl2/3-MMP* silencing, and the co-localization of P69B and Sl2/3-MMP in the apoplast, indicated that P69B might be a substrate. P69B indeed contains an MMP cleavage site and can be cleaved by Sl2/3-MMP *in vitro*, consistent with being a substrate. In addition, the induction of cell death upon MMP silencing was reduced in P69B-silenced plants, suggesting that MMP and P69B act in the same signalling cascade controlling cell death. However, cleavage of P69B by Sl2/3-MMP inactivates P69B and causes its degradation, not its activation (Zimmermann *et al.*, 2016). Thus, although the MMP–P69B interaction fulfils criteria (2), (3), and (4), it does not fulfil criterion (1) and therefore does not represent a proteolytic cascade.

### Does γVPE activate AtCPY in Arabidopsis?

Vacuolar processing enzymes (VPEs) are vacuolar Cys proteases from the C13 family also known as legumains or asparaginyl endopeptidases (AEPs). VPEs specifically cleave after Asn and Asp residues, and are thought to regulate many vacuolar proteins by processing. Amongst the proposed substrates is carboxypeptidase Y (AtCPY, family S10, SCPL48). Indeed, in Arabidopsis mutants lacking γVPE, the Arabidopsis homologue AtCPY accumulates at a higher molecular weight when compared with processed AtCPY in wild-type plants, and this processing is restored when expressing γVPE in the *γvpe* mutant (Rojo *et al.*, 2003). These data confirm that γVPE indeed processes AtCPY *in planta*. However, what is the effect of this processing on AtCPY activity? The accumulating AtCPY in *γvpe* mutant plants corresponds to an activated version, based on its molecular weight. CPY in *Schizosaccharomyces pombe* is cleaved twice: once to remove the autoinhibitory C-terminal prodomain, activating the protease, and a second time inside the protease domain, resulting in a disulphide-linked heterodimer (Mukaiyama *et al.*, 2011). In Arabidopsis, it is probably the second cleavage in AtCPY that is caused by VPE. In other words, VPE converts the already active AtCPY into an active heterodimer. Conclusive evidence for this hypothesis would require *in vitro* cleavage experiments and activity assays of ProAtCPY with VPEs. Therefore, although the VPE–CPY interaction fulfils criteria (2), (3), and (4), it probably does not fulfil criterion (1) and does therefore not represent a proteolytic cascade.

### Does VmPE-1 activate SH-EP in urad beans?

SH-EP is a vacuolar papain-like protease (family C1A) synthesized in cotyledons of germinating *Vigna mungo* seeds (urad beans). SH-EP is responsible for degradation of the seed proteins accumulating in protein storage vacuoles. Like other papain-like proteases, SH-EP can be activated autocatalytically *in vitro*, but this process is slow and pH dependent (Okamoto *et al.*, 1999). When added to proSH-EP *in vitro*, VmPE-1 (*V. mungo* processing enzyme 1, a VPE-like C13 protease) catalyses SH-EP activation at pH 6 where autocatalytic activation of SH-EP is restricted (Okamoto *et al.*, 1999). The pH dependency highlights the complex nature of protease activation. The accumulation of proSH-EP in plants lacking VmPE-1 [criterion (4)] has not yet been reported. The authors highlight that even if SH-EP can autoactivate *in vitro* under suitable conditions, these autocatalytic mechanisms might not function *in vivo*, and that other proteases such as VPEs may accelerate the activation of SH-EP at pH conditions unfavourable for autocatalytic activation.

### Does VPE activate RD21 in *A. thaliana?*

RD21 from *Arabidopsis thaliana* (At1g47128) is an abundant plant-specific papain-like Cys protease (family C1A) carrying a C-terminal granulin domain. RD21 maturation starts with the proteolytic removal of the N-terminal prodomain, resulting in the generation of an ‘intermediate’ form of the protease. This is followed by the removal of the C-terminal granulin domain, producing the ‘mature’ form of the protease. Interestingly, RD21 produced in insect cells cannot mature itself, unless it is mixed with a leaf extract (Yamada *et al.*, 2001). Likewise, the prodomain of the catalytic mutant of RD21 is still removed when expressed in *Nicotiana benthamiana* (Gu *et al.*, 2012). However, a bimolecular activation by RD21 or its orthologues remains to be excluded. These findings indicate that RD21 is activated by other proteases and is therefore part of a proteolytic cascade. RD21 was suggested to be a substrate of VPEs (Yamada *et al.*, 2001). It is unclear, however, where within ProRD21 VPE would cleave. In addition, even though these proteases are co-expressed and co-localize in the vacuole, RD21 is still fully matured in *A. thaliana vpe* quadruple plants lacking all four VPEs (Gu *et al.*, 2012). Therefore, although the VPE-RD21 proteases fulfil criteria (1), (2), and perhaps (3), they seem not to fulfil criterion (4) and are therefore not a proteolytic cascade. Furthermore, in contrast to Arabidopsis RD21, both the RD21 ortholog from *N. benthamiana* (CypP6) and a homologue from a different subfamily (CP14/NbC14) can activate themselves upon heterologous expression (Paireder *et al.*, 2016, 2017), indicating that the RD21-activating pathway may not be conserved between plant species.

### Does MC9 activate RD21-like proteases in poplar?

The metacaspase-9 homologues PttMC13 and PttMC14 from *Populus* were proposed to process an RD21-like protease during xylem formation in *Populus* trees (Bollhöner *et al.*, 2018). This conclusion is based on quantitative proteomics data where peptides derived from the prodomain region of the RD21-like protein were more abundant in PttMC13- and PttMC14-silenced plants when compared with wild-type plants, indicating that PttMC13 and/or PttMC14 process the RD21-like protease [criterion (4a)]. However, peptides located in the peptidase domain of the RD21-like protease did not seem to differ in abundance between the lines, suggesting that the precursor of the RD21-like protease does not accumulate [criterion (4b)]. Also no signiﬁcant difference in the abundance of the RD21-like protease was found in PttMC13 overexpression lines, suggesting that the MCs are not rate limiting in this process. Although PttMC13 and PttMC14 have a predicted cytonuclear localization, and RD21-like proteases are usually vacuolar, co-localization of the proteases could theoretically occur after tonoplast rupture. In Arabidopsis, RD21 was not reported amongst the substrates of MC9 in Arabidopsis (Tsiatsiani *et al.*, 2013) and the RD21 prodomain seems to be cleaved at a site that does not match the MC9 substrate preference (Vercammen *et al.*, 2006; Gu *et al.*, 2012). Taken together, the MC9–RD21 cascade is based on circumstantial evidence and remains to be confirmed. The proteomics data in the report on PttMC14 and PttMC14 also indicate that these proteases might activate an Asp protease (family A1), but this was not investigated further.

### Does an RD21-like protease activate aleurain in barley?

Aleurain is a Cys aminopeptidase of the C1A family located in the lytic vacuole. Aleurain maturation has been intensively studied in barley (Holwerda *et al.*, 1990). Proaleurain produced in *Xenopus* oocytes is not capable of self-activation, consistent with its capacity as an aminopeptidase (Holwerda *et al.*, 1990). However, this recombinant proaleurain is processed upon addition of aleurone cell extracts (Holwerda *et al.*, 1990). A protease that activates recombinant proaleurain was isolated from cauliflower florets and this was an RD21-like protease carrying a C-terminal granulin domain (Halls *et al.*, 2005). Likewise, purified pro-aleurain from *N. benthamiana* (NbALP) can be activated *in vitro* by NbC14, an RD21-like protease (Niemer *et al.*, 2016). However, in Arabidopsis *rd21* knock-out mutants, the aleurain-like protease AALP is still matured, demonstrating that RD21 is not required for AALP activation in this plant species (Gu *et al.*, 2012). Taken together, these studies show that aleurains are activated by proteases, possibly by RD21-like proteases, but this hypothesis remains to be confirmed.

## Discussion

While well described in animal biology, proteolytic cascades represent an uncharted territory in plants. None of the plant studies above has established a hierarchical, activating relationship between two or more proteases. Plants do not have caspases or coagulation factors, so no direct analogies can be drawn from described cascades in animals. However, plants do not have fewer proteases compared with animals. Extended plant protease families such as subtilisins and pepsins might have evolved to participate in proteolytic cascades in plants.

Plants also have similar requirements for quick and local regulation of proteolytic activity, for example in pathogen defence, wounding, PCD, and development. For instance, latex coagulation upon wounding is quick, and the latex of papaya contains high concentrations of Cys proteases (El Moussaoui *et al.*, 2001). The low proteolytic activity in fresh latex suggests that the majority of proteases are only activated upon wounding (Silva *et al.*, 1997). Likewise, the latex of the unrelated rubber tree *Hevea brasiliensis* contains hevein, which is proteolytically released from its precursor and acts as coagulant (Lee *et al.*, 1991). Proteolytic cascades are very likely to occur here, but remain to be demonstrated.

There are several reasons why proteolytic pathways are elusive in plants. First, plants are very diverse and have many specialized features, so proteolytic pathways may not always be conserved across different plant species. Secondly, post-translational modifications such as those in proteolytic cascades are not necessarily regulated at the transcriptional level. Thus, commonly used gene expression platforms such as RNA sequencing will not identify proteolytic cascades because they are sparked as an immediate, local response to stress using proteases and substrates that are already present in their inactive, unprocessed form. In addition, we will miss proteolytic cascades because the classic time points chosen in plant studies are often hours or days after the treatment, which is too late to detect post-translational modifications including early proteolytic events. Fourthly, redundancy in plant protease families might hamper classical genetic screens. Remarkably, several proteolytic cascades have been identified in animals using classical genetic screens. However, despite the powerful genetic screens in plant science, these cascades have not been discovered by genetics. For instance, genetic screens identified SDD1, a subtilisin-like Ser protease involved in the regulation of stomatal density (Berger and Altmann, 2000). However, no additional proteases have been identified in these screens, either because SDD1 does not act in a proteolytic cascade, or because of the high protease redundancy in plants.

However, despite these limitations, several technological advances could uncover proteolytic cascades in plants in the near future. Advanced proteomics techniques such as TAILS and COFRADIC (Gevaert *et al.*, 2006; Kleifeld *et al.*, 2010, 2011) will help to identify proteases amongst the substrates of key proteases. Furthermore, dynamic protease activation events can now be monitored using activity-based protein profiling (Morimoto and van der Hoorn, 2016). It is now also possible to overcome redundancy in the protease family with the advance of genome editing techniques and tissue-specific expression of protease inhibitors (Schardon *et al.*, 2016). Finally, heterologous expression of the protease precursors (e.g. Holwerda *et al.*, 1990; Yamada *et al.*, 2001; Niemer *et al.*, 2016) has been instrumental to study protease activation, and similar approaches will be useful to discover proteolytic cascades in the future.
